# A Review of COVID-19-Related Literature on Freight Transport: Impacts, Mitigation Strategies, Recovery Measures, and Future Research Directions

**DOI:** 10.3390/ijerph191912287

**Published:** 2022-09-27

**Authors:** Ahmed Karam, Abdelrahman E. E. Eltoukhy, Ibrahim Abdelfadeel Shaban, El-Awady Attia

**Affiliations:** 1 Department of the Built Environment, Aalborg University, 9220 Aalborg, Denmark; 2 Department of Mechanical Engineering (Shoubra), Benha University, Benha 11672, Egypt; 3 Department of Industrial and Systems Engineering, The Hong Kong Polytechnic University, Hong Kong SAR, China; 4 Mechanical and Aerospace Engineering Department, UAE University, Al-Ain P.O. Box 111, United Arab Emirates; 5 Department of Industrial Engineering, College of Engineering, Prince Sattam bin Abdulaziz University, Al Kharj 11942, Saudi Arabia

**Keywords:** freight transport, COVID-19, air freight, sea shipping, road transport, recovery measures, strategies

## Abstract

The COVID-19 pandemic has caused significant disruptions in the freight transport sector. The number of studies on the impact of COVID-19 on freight transport and possible mitigation strategies are growing. However, a systematic and comprehensive review highlighting the research themes, main findings, research methods, and future research directions of these studies remains scarce. Therefore, this study presents a mixed review comprising scientometric and systematic reviews to cover these research gaps. Results show that 68 studies have been published on this topic since the beginning of 2020 and that they cover three main themes: the impacts of COVID-19 on freight transport, mitigation strategies, and recovery during and after COVID-19. In addition, we describe the research methods, main findings, and possible research directions in each of them. Thus, the findings of our work present both theoretical and practical analyses of COVID-19-related research on freight transport and provide important future research directions in this domain.

## 1. Introduction

In December 2019, China announced the first case of the novel coronavirus disease, called “SARS-CoV2”. Since then, the virus has spread over the world, infecting and killing people. By March 2020, the World Health Organization (WHO) had declared it a pandemic and officially named it “COVID-19”. By the end of 2020, the virus had infected more than 82 million people and killed over 1.8 million. Therefore, the pandemic may be referred to as the most disastrous black swan event in 2020. To curb the spread of COVID-19, travel restrictions were firmly implemented by governments worldwide. As a result, these restrictions have significantly affected all modes of freight transport and caused severe disruptions. As of April 2020, global demand for air freight has dramatically declined by approximately 27.7% compared to 2019 [1], while air freight capacity shrank by 42% compared to 2019 [2]. This dire situation has also been observed in global road freight, with an estimated loss of EUR 550 billion due to movement restrictions imposed by governments [3]. Similarly, sea freight transport was not spared, as container ship mobility reduced by approximately 14% in June 2020 [4].

Freight transport has always been an important aspect of the supply chain. Therefore, since 2020, many studies have studied the impact of COVID-19 on freight transport logistics and possible mitigation strategies. Examples of these impacts include shortage of transport resources [5,6], higher operating costs [7,8], and fluctuations in demand for transport services [6,9]. The identification of appropriate strategies for managing such impacts has been addressed in some studies [10,11]. Given the significance of this topic, it is valuable to report the up-to-date status of research regarding freight transport during COVID-19 and outline some future research directions. It is worth noting that some previous studies have addressed the impact of other types of pandemics, e.g., influenza [12], cholera [13], Ebola [14], and malaria [15]. In addition, some scholars have published review papers on the impacts and response strategies to these disease outbreaks [16,17]. However, these review papers are focused on the pre-COVID 19 pandemics with emphasis on logistics [16], supply chains [17], and buying behaviors of customers due to pandemic outbreaks [18]. Compared to the previous pandemics, COVID-19 stands out with extraordinary impacts on virtually all human activities. Unlike the previous pandemics, that disrupt activities in some locales, COVID-19 affects businesses around the globe. Furthermore, to curb the spread of the disease, many countries implement lockdowns that halt all industrial and social activities with attendant disruptions to most global and local supply chains [19]. Although the COVID-related literature on freight transport is constantly growing, the findings from these studies are still scattered across several sources and rather unsystematized. More specifically, the literature reporting COVID-19-pandemic-related freight transport studies in a systematic approach remains scarce. In this regard, there are only two relevant review papers [19,20]. Chowdhury, Paul, Kaisar and Moktadir [19] conduct a review study on the impacts of the COVID-19 pandemic on eight supply chain-related areas including transportation and logistics. Despite the significance of this review study, it considers only a small set of the COVID-19-related freight transport studies, i.e., 12 studies. Borca, Putz and Hofbauer [20] review the literature on the different crises that hit Europe in the previous 20 years and their effects on freight transport modes. Although the authors list COVID-19 as one of the crises, only three studies related to the impact of COVID-19 on freight transport are covered.

Unlike the previous studies, this current work analyzes 57 COVID-19-related studies on freight transport. In particular, this work seeks to answer the following three main research questions (RQ):

RQ1: What are the research themes and main findings of the literature on freight transport during COVID-19?

RQ2: What are the main research methods used in the COVID-19-related literature on freight transport?

RQ3: What are the future research directions in the freight transport sectors with respect to the COVID-19 pandemic?

To answer these three research questions, the present study uses a mixed review method that combines scientometric and systematic review methods. A scientometric analysis is conducted to identify the major research scopes, linkage of researchers, countries, and others on research articles related to COVID-19 and freight transport. Next, a systematic review of these articles is undertaken to summarize the main research themes in this domain. The findings of this work will be of immense benefits to researchers and forestall duplication of research efforts at this stage of the pandemic. Additionally, researchers can take cues from the identified future research avenues to enrich the existing body of knowledge in the domain of freight transport. Furthermore, practitioners will gain deep insight into the main themes of freight transport and how scholars have addressed them. This will help bring more innovation and performance improvement in this field.

The remainder of this paper is organized as follows. Section 2 presents the review methodology for scientometric analysis and systematic review. In Section 3, results of the scientometric analysis are presented, whereas Section 4 discusses the systematic literature review. Lastly, Section 5 concludes the study.

## 2. Review Methodology

**Figure 1:**
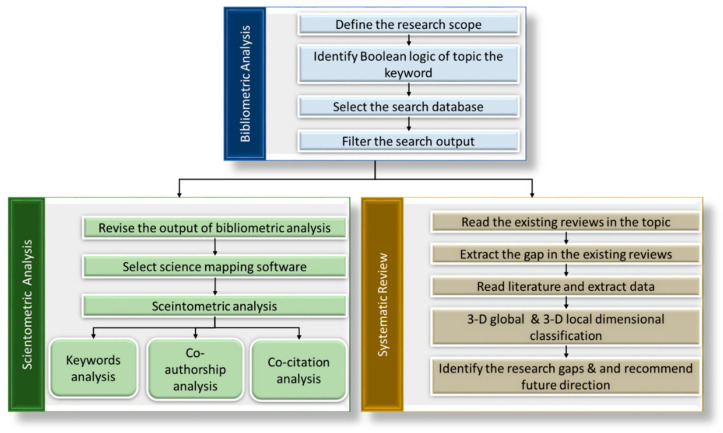
Summary of the review methodology.

Figure 1 summarizes the methodology of the review process, which combined bibliometric analysis, scientometric analysis, and systematic review. Note that the bibliometric analysis was conducted before the scientometric analysis and systematic review, to ensure that the collected bibliographic data were representative of the proposed review topic. The following sub-sections expatiate on the details of each process.

### 2.1. Bibliometric Analysis

**Figure 2:**
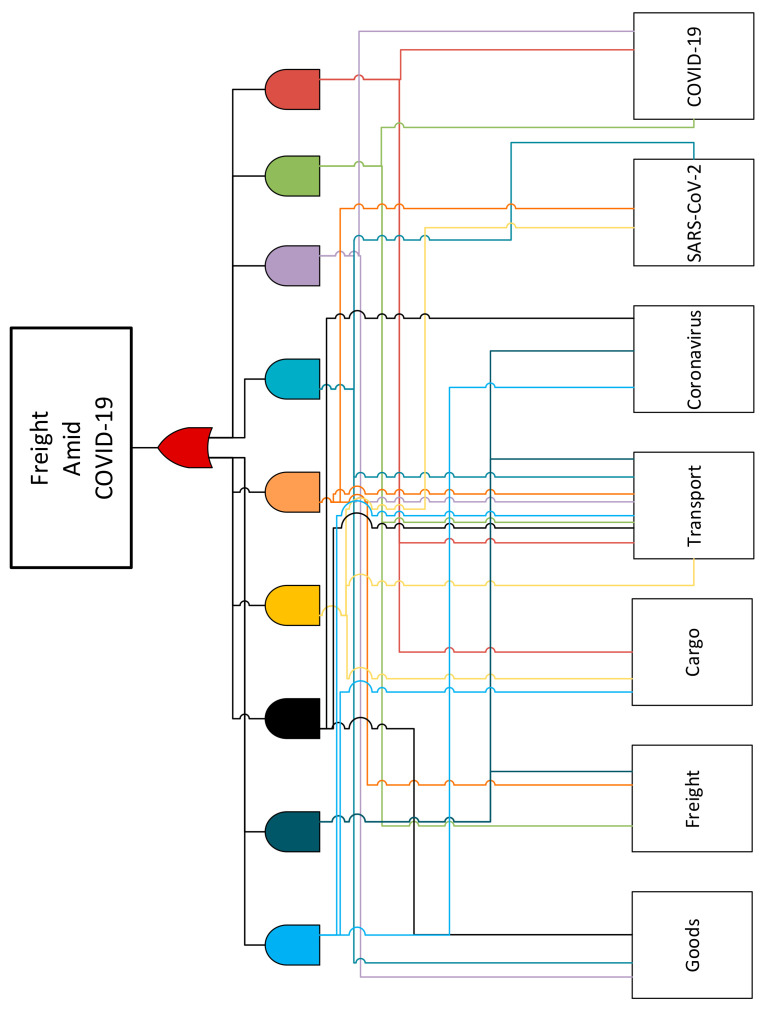
Keywords combination through Boolean logic gates.

The bibliometric analysis started with the definition of the scope, which was freight transport amid the outbreak of COVID-19. Next, based on this scope, combinations of three keywords and their related synonyms were used to search for the related literature in a scientific database. These keywords, “COVID-19, coronavirus, and SARS-CoV-2”, “transport”, and “freight, cargo, and goods”, were combined by using Boolean logic, as shown in Figure 2. After generating the combination of keywords, the SCOPUS database was selected due to its updated and wide coverage of existing literature on the proposed topic [21]. The search results gave 101 research documents, which further dropped to 57 articles when only English language documents were extracted. Note, the search results were downloaded in a CSV file, which included each article’s title, abstract, author list, funding details, etc. The bibliometric analysis ended with the filtering process, which comprised screening the articles’ titles and reading their abstracts to eliminate irrelevant research papers.

### 2.2. Scientometric Analysis

Scientometric analysis is a data and text mining technique that uses the filtered data from the bibliometric study to visualize the relationships between keywords, citations, authors, and others. This analysis reduces the subjectivity associated with the findings of narrative reviews [22]. Thus, this analysis is adopted in several studies in different research fields, including transportation [23], construction management [21], and healthcare management [24]. As shown in Figure 1, the first step in conducting the scientometric analysis was to revise the results of the bibliometric analysis and then select a suitable software to perform the scientometric analysis. In this study, VOSviewer [25] was utilized for the scientometric analysis, including analysis of keyword co-occurrence, co-citation, and co-authorship.

### 2.3. Systematic Review

**Figure 3:**
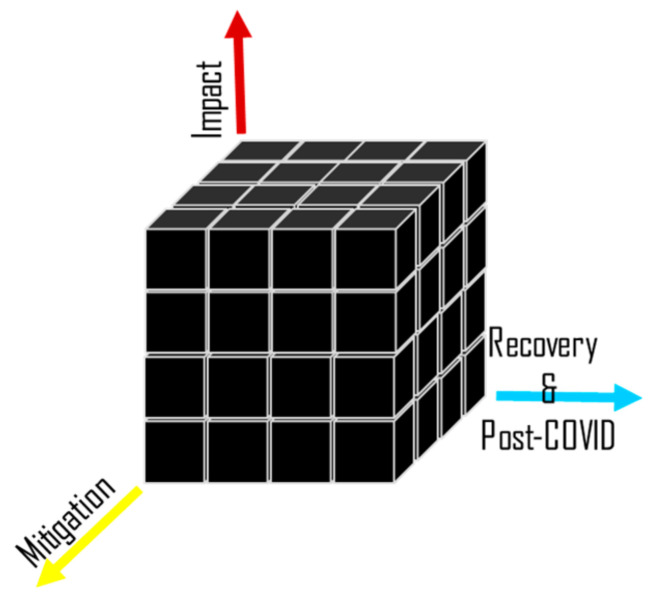
Schematic diagram illustrating the 3-D matrix research themes, including impact, mitigation, and recoverability.

Despite the advantages of the scientometric analysis, it is still insufficient to provide a comprehensive understanding of the bibliometric results. Therefore, a systematic review can be deployed to organize the extracted articles based on a number of research themes. As shown in Figure 3, we identified three broad research themes in the existing studies. These included the impact of COVID-19 on freight transport, the mitigation strategies that were adopted, and the ability to recover during and after COVID-19. At the early stage of the pandemic, countries of the world proposed several measures to fight the spread of infection, which in turn influenced freight transport systems in several ways, as reported by researchers and governmental reports. We identified these studies and categorized them under five main impacts. During the pandemic or what is now called the new norm, maintaining food and medical supplies was dependent on creating robust freight transport systems. To this end, practitioners paid great attention to risk mitigation programs, researchers proposed novel mitigation strategies based on state-of-the-art technologies, and additionally, some regulations on freight transport were relaxed in many countries. Some of these mitigation strategies are expected to lead to rethinking the future of freight transport in the post-pandemic era. We categorized these mitigation strategies into five, which included (1) usage of autonomous delivery vehicles (ADVs), (2) drone delivery, (3) deployment of mobile warehouses, (4) engagement of large ships, and (5) application of quantity discounts. In addition to adopting risk mitigation strategies, the evaluation of the recoverability of freight transport systems was tackled through several measures, such as system recovery rates, quality, demand recovery, and efficiency.

## 3. Results and Discussion of the Scientometric Analysis

Table 1 summarizes the selected scientometric analysis performed in this study, including keyword co-occurrence, co-authorship, and citation analysis. For each analysis, its related criteria are fixed, as shown in Table 1. Based on the search output from the SCOPUS database, the 57 documents that cover the research scope contain 604 keywords. To study the most co-used keywords in these documents, a threshold criterion of three is applied (i.e., the minimum occurrence of a keyword is set to three). Forty-two keywords meet the threshold criterion, and all of them are connected. In analyzing co-authorship between 40 countries of the authors who have published on freight transport during COVID-19, two thresholds are applied: one for the minimum number of documents (i.e., 2) per country and another for the minimum citations (i.e., 0) of countries. Eighteen countries meet the threshold criteria, and only fourteen of them are connected. In the following sub-sections, the results of these analyses are discussed. Similarly, in the citation analysis of the sources, 36 sources meet the threshold criteria, but only 13 are connected.

### 3.1. Keyword Analysis

**Figure 4:**
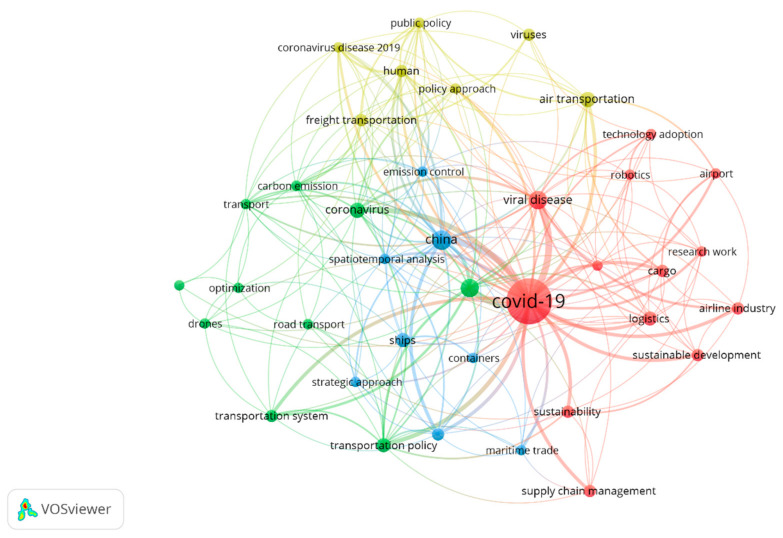
Keyword mapping network.

Keyword analysis is used to generate the keywords mapping network shown in Figure 4. The mapping network consists of nodes connected by lines. Keywords with bigger nodes mean they are more frequently used than others with smaller nodes. Similarly, a thicker connecting line between two keywords indicates the frequency of their occurrence in multiple articles. Therefore, the keyword “COVID-19” is the most frequently used. Meanwhile, it co-occurs most with the keywords “Airline industry”, “Maritime trade”, “Transportation policy”, etc. This implies that most of the research in this domain focuses on policies aimed at mitigating the impact of the COVID-19 pandemic on freight transportation.

Table 2 shows the top 15 keywords used in freight transport studies amid COVID-19, while others are shown in the keyword mapping network in Figure 4. For instance, “drones” can be regarded as one of the most important mitigation strategies, though further investigations into their use are required. This is because of the legal differences to fly them in different countries [26]. Additionally, COVID-19 can be deemed as one of the long-term disruptions. For example, flight schedules are interrupted, borders are closed, and work modes have changed. However, the word “optimization” is not used sufficiently to cope with the disruption in the three modes of freight transport.

### 3.2. Co-Authorship Analysis

**Figure 5:**
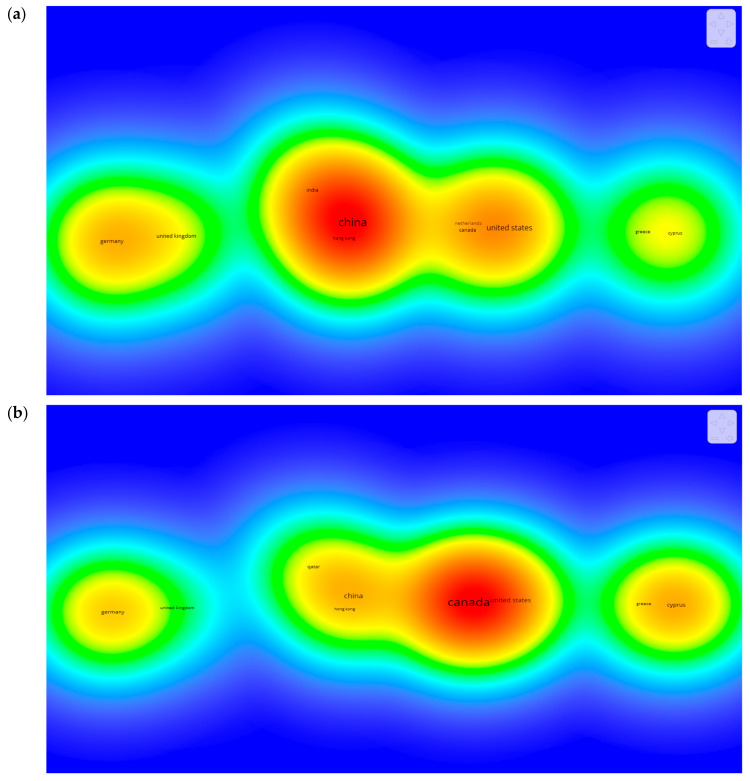
Density visualization of the co-authorship between countries based on (**a**) total published documents, and (**b**) number of citations.

As COVID-19 has spread worldwide, it is interesting to see how different countries cooperate in the research on freight transport. For this purpose, a co-authorship analysis is conducted, as shown in Figure 5. Figure 5a shows that the highest co-authorship in terms of the number of published research works is found in the cluster comprising China, Hong Kong, and India. However, in Figure 5b, the cluster of the most cited authors includes Canada and the United States. This means that there is no direct correspondence between citation and number of publications.

### 3.3. Source Citation Analysis

The number of citations of an article does not only reflect its impact but also affects the impact of the publishing source. Thus, the analysis of citation is necessary to identify the most impactful research works, sources, and authors [22]. However, Table 3 shows that the journal sources with a high number of research articles do not necessarily publish high impact research articles with high citation counts. For more detail, until the time of our research (the last update of these scientometric data was on 11 December 2021), *Sustainability* (Switzerland) and *Science of Total Environment* (SOTE) have received 39 and 37 citations, respectively. However, these citation counts do not reflect the exact impact of these two journals, because the number of documents published by *Sustainability* and SOTE are 10 and 1, respectively. This means that the average citation for *Sustainability* does not exceed 4 compared to 37 in SOTE. Moreover, normalization of citation counts is a metric that can be used to eliminate the effect of time on source citation (i.e., the propensity that an older document is more likely to get higher citations than a newer one). For example, the average number of citations for *Transportation Research Interdisciplinary Perspectives* (TRIP) is 30, while it is 79 for the *International Journal of Advanced Manufacturing Technology* (IJAMT). However, the normalized citation of TRIP is 3.7687, which is almost three times the normalized citation of IJAMT. To conclude, the most impactful journal within the period under review is the *Canadian Journal of Agricultural Economics,* because it has the highest average citations and the highest normalized citation.

## 4. Results and Discussion of the Systematic Review

### 4.1. COVID-19-Related Impact

Our review shows that 36 out of the identified studies investigate different impacts of COVID-19 on the freight transport industry. These 36 studies are carefully analyzed to identify and group the different impacts of COVID-19 on freight transport. There are five categories of these impacts, including impact on: (1) transport demand, (2) transport capacity, (3) operational transport cost, (4) delivery performance, and (5) carbon emissions, as shown in Table 4. It should be noted that a study may report more than one impact; therefore, the sum of all entries under ‘studies per impact’ is not necessarily 36. In the following subsections, these 36 articles are classified and thoroughly analyzed based on the reported impacts, transport mode, and research methodology.

#### 4.1.1. Impacts of COVID-19

- Demand for freight transport services:

Twenty-four studies report the impact of the COVID pandemic on the demand for different freight transport modes, i.e., air, maritime, rail, and road. Six studies examine the impact of COVID-19 on the demand for seaports and waterways transport [6,7,9,37,39,40,41,42,43]. Tianming, Erokhin, Arskiy and Khudzhatov [9] show that the pandemic breaks the connectivity among seaports in China, Norway, Iceland, Russia, South Korea, and Sweden. In Australia, Munawar, Khan, Qadir, Kouzani and Mahmud [6] report that during the initial stage of the pandemic, the demand for water freight transport declines by 9.5%. Michail and Melas [7] indicate that the daily number of calls of dry bulk and clean tankers in ports is highly affected by the pandemic, while the pandemic has no impact on the calls for crude oil vessels. Narasimha, Jena and Majhi [37] confirm that the overall demand for major Indian seaports declines by 4.59% for 2020 compared to 2019. A case study of a maritime transport service provider shows that the transshipments of cargo decreases by approximately 32%, whereas the urgent supplies of medical equipment increase [39]. Further, five studies examine the impact of the COVID-19 pandemic on the demand for airfreight transport [5,6,34,36,38]. For example, in Australia, the demand for airfreight transport decreases significantly from 84.8 million kilograms in January 2020 to 54.5 million kilograms in April 2020 [6].

Nine studies examine the impact of the COVID-19 pandemic on the demand for road freight transport [6,8,11,20,27,28,29,30,33]. For example, Arellana, Márquez and Cantillo [28] show that freight trips in Colombia reduce by around 38% due to a low demand for non-essential products. Due to the lockdown and travel restrictions, people shift from in-store shopping to online shopping and demands for last-mile deliveries increase [6,8,11,27]. From January 2020 to June 2020, global retail platforms witness a 26.9% increase in online-shopping orders [57]. In Europe, online shopping sales in April 2020 increase by 30% compared to April 2019 [58]. Due to the panic buying behavior of people during the pandemic and the sharp fall in oil prices, Ho, Xing, Wu and Lee [33] report a positive correlation between road freight transport turnover and the number of confirmed cases of COVID-19 in China. In Portugal, Cruz and Sarmento [30] state that during the peak of COVID-19 in April 2020, the traffic volume of light commercial vehicles reduces by an average of 44.5%, while the traffic of heavy trucks reduces by 24%. Bartuska and Masek [29] conclude that the traffic volumes of trucks and trailers are not significantly affected on the major roads of South Bohemia (Czech Republic). Borca, Putz and Hofbauer [20] investigate road transportation in the USA at the beginning of the pandemic. They report that despite an unprecedented 46% reduction in the traffic volume of private cars, freight traffic volume reduces by a mere 13%. These values of commercial traffic volumes indicate that the supply of essential products is not significantly disrupted, especially to supermarkets and pharmacies during the pandemic. Additionally, this also implies that many companies, even if closed, increase their inventories to be safe against future uncertainties and to start production again once the lockdown ends. Although the previous studies focus on a single freight mode, some studies investigate multiple freight modes. For example, Cui, He, Liu, Zheng, Wei, Yang and Zhou [31] study the impacts of the COVID-19 pandemic on China’s transport sectors. Their results show a reduction in the outputs of households and production sectors; thus, there is a reduction in the demand for transportation in these sectors. For example, aviation freight transportation reduces by 2.81%, while road freight transportation, railway freight transportation, and waterway freight transportation are down by 2.20%, 1.84%, and 1.04%, respectively. Gray [32] reports an increasing demand in the agriculture sector for different transportation services, e.g., bulk ocean freight, rail movement, and road trucks. Li, Bai, Hu, Yu and Yan [42] present equations to evaluate the traffic variations of overall provincewide traffic and tested the hypotheses for traffic recovery analysis. The results show that truck traffic dropped by 68.19% during the outbreak and found that the higher the GDP per capita the region has, the more the traffic in the region was affected by the pandemic. It is also found that truck traffic had a rapid recovery period compared to that of passenger traffic.

- Capacity shortage

Ten studies report the impact of the COVID pandemic on the transport capacity of the different freight transport modes. The imposed epidemic control requirements lead to a shortage of transport capacity, e.g., driver shortage, belly-hold shortage in airfreight transport, and shortage of empty containers. In April 2020, passenger flights decrease by approximately 95% compared to the previous year [45]. Similarly, belly capacity for international air cargo drops by 75% in April 2020, compared to the previous year. Meanwhile, at the peak of COVID-19, the demand for medical and personal protective equipment significantly increases worldwide. On the other hand, air freight capacity is severely limited due to the shutdown of passenger flights [5,6,34,48] and the available cargo aircraft cannot resolve the shortage in airfreight capacity. To resolve this, passenger aircraft are granted exemptions from July 2020 to July 2021 to carry freight in the main cabin when no passengers are being carried [59].

Workforce capacity of the transport sector has also been shown to be vulnerable to shortage because of mobility restrictions and sickness [45]. For instance, truck drivers cannot access many toilets at rest areas since they are closed. Therefore, mobile toilets are provided for truck drivers in Canada [32]. In China, the beginning of the pandemic coincides with the holiday season, when most logistics laborers have travelled to their hometowns. Subsequent restrictions on people’s mobility result in a severe workforce shortage in the logistics industry [35]. On the bright side, quarantine obligations lead to the high availability of some categories of the workforce, e.g., truck drivers, in freight transportation [46]. For example, most truck drivers working in Western European countries, e.g., France, Denmark, and Germany, are from Eastern European countries, e.g., Poland and Romania. To avoid workforce shortages during the holiday periods, logistic companies request their drivers to postpone their holidays to avoid the mandatory 14-day quarantine.

Containerized trade represents 90% of the global trade volume. Therefore, containers are essential resources to transport different products between countries. In the container supply chain, containers are frequently returned empty to the exporting country. The COVID-19-related shutdown of many production facilities worldwide and the subsequent reduction in demand result in a shortage of empty containers [47]. Some food products, transported in containers, are delayed due to lack of empty containers in North America [32]. In addition, shortage of truck drivers, stringent inspection protocols, and requirement for quarantine delay the delivery of maritime freight [48]. Compared to sea and road transport, rail transport is less labor-intensive since the rail transport sector relies on a small size of very specialized crews to operate large trains. However, rail transport may have been impacted by COVID-19 if a significant proportion of the very specialized workforce is infected [32].

- Operating cost and prices of transport services

Thirteen studies report the impact of the COVID-19 pandemic on the operating costs of freight vehicles. The reviewed literature identifies three reasons responsible for the increase in operating costs of the logistics industry in China. Firstly, restriction on people’s mobility cause a shortage in workforce capacity, leading to higher labor costs and freight rates [5,32,35,49]. Secondly, long-term infection prevention, e.g., disinfecting parcels and contactless delivery facilities, attract extra cost [31,34,35]. Thirdly, uncertainties in labor availability, traffic restrictions, and status of pandemic outbreaks incur extra costs in planning transportation routes [7,27,35]. Gray [32] reports that COVID-19 prevention and protective measures (e.g., multiple drivers are prevented from using the same truck) have led to increased transport costs of agricultural products in Canada. To support companies in applying such measures, Canadian transport authorities increase the maximum hours of service for drivers [60]. Some logistics enterprises adopt new modes of logistics services to fight the COVID-19 pandemic through the use of contactless delivery. This has also increased their operating costs, since contactless delivery needs extra investment cost in contactless facilities [8]. Michail and Melas [7] show that an increase of 1% in the global COVID-19 infection cases decrease the freight rates of Baltic dry and dirty Tankers by 0.03% and 0.046%, respectively. Bartle, Lutte and Leuenberger [5] report that the airfreight rates increase during the pandemic due to limited air cargo capacity. For example, in June 2020, the airfreight rates from China to Europe are 254% higher than those of June 2019 [50]. By using a multi-sectoral computable general equilibrium model of China, Cui, He, Liu, Zheng, Wei, Yang and Zhou [31] estimate that the cost of protective measures (e.g., disinfection, quarantine, and parking inspection) lead to a 1.5% reduction in the production efficiency of the freight transport sector. Grzelakowski [52] found that during the first months of 2020, the global maritime shipping sector, and specifically container transport, was affected by the lockdown and the resulting congestion in port terminals because of the inability of carriers to pick up freight from seaports and, thus, cargo ships became floating warehouses. In the middle of the third quarter of 2020, the supply chains become more stabilized, the demand on freight transport increased and, consequently, the spot freight rates also increased significantly. This was apparent in container shipping at the Far East–USA trade lane, as well as the routes towards South America or West Africa and the Middle East–Western Europe trade lanes.

On the bright side, COVID-19-related restrictions have led to a reduction in fuel prices. On 20 April 20 2020, the price of West Texas Intermediate crude oil dropped by 42.5% [61]. Fuel price accounts for approximately 15 to 20% of the total operating cost of the transport industry. The IATA reports that in July 2020, fuel prices are 45% lower than the price during the same period in 2019, resulting in a saving of nearly USD 70 billion for the airline industry [51]. Regardless of the drop in oil prices, travel restrictions and low demand for transport services result in a marginal profit for freight transport providers [6]. Some countries adopt policies, e.g., tax reduction, to reduce the operating costs of logistics companies. For example, China exempted logistics enterprises from vehicle tolls and value-added taxes on income due to delivering essential living supplies [8].

- Delivery performance

Nine studies report the impact of the COVID pandemic on the delivery performance of different freight transport modes. COVID-related restrictions exert the most impact on the delivery performance of maritime and air transport sectors. This is because these two sectors apply some quarantine virus containment measures to shipments, vessels, and crew. Several importing countries impose a 14-day quarantine for containers upon arrival at the port, resulting in a lengthy cycle times of containers [32]. In addition, COVID-19-related shortage of transport capacities, e.g., workforce, vehicle capacity, and empty containers, increase the delay in freight delivery [47,48]. Koyuncu, Tavacioğlu, Gökmen and Arican [54] suggest that the COVID-related restrictions on production and trade may break the interconnected supply chain; thereby, serious losses might be faced in some container lines.

Due to restricting people’s mobility, several cities worldwide witness reduced road traffic. Therefore, some studies report positive effects on road freight transport, such as fewer traffic jams, increased efficiency, and fewer emissions [20]. For example, until the so-called “new normal” (11/05/2020), road traffic drops, on average, down to 76.28% in Madrid [27], 73% in UK [62], Sanpaolo (−55%), New York (−74%), Milan (−74%), Barcelona (−73%), Stockholm (−48%) [40]. The reduced road traffic causes delivery times to be much shorter [6]. Since the pandemic reduces demand for all nonessential products, access of essential products to seaports, rails, and trucks has generally improved [32]. However, finding truck parking is more difficult, especially for a large fleet [53]. This is because most employees are working from home and their private cars are occupying the parking slots in the streets. During the pandemic, many shops, e.g., pharmacies, grocery stores, and bakeries, start pickup and home delivery options in an attempt to stay in business. Thus, the efficiency and quality of last-mile logistics services are impaired [8,55].

- Emissions

Four studies report the impact of COVID-19 on the emissions from different freight transport modes. The COVID-19 pandemic creates an opportunity for achieving some temporary sustainability gains in the transport sector. This is mainly due to the reduction in carbon emissions, mainly because of mobility restrictions. However, this might be offset by increasing demand for cargo transport [5]. Few studies report the reduction in CO_2_ emissions from freight transport during the pandemic [5,28,38,56]. In China, COVID-19 significantly reduces industrial and transport activities, resulting in lower CO_2_ emissions. During the first quarter of 2020, freight transport has a 22.3% reduction in CO_2_ emissions, while passenger transport is 59.1% compared with the same period of 2019 [56]. In Columbia, mobility restrictions have created positive side effects regarding externalities of transport, such as less traffic safety issues, noise, and pollution [28]. In Croatia, some airports have an average reduction of 96% in flights in April 2020 compared to April 2019. This directly affects the reduction in CO_2_ emissions by a factor of 1.81 for the commercial airport and 3.49 for the seasonal airport [38].

#### 4.1.2. Transport Mode

Table 5 classifies the 32 studies based on transport mode. Table 5 shows the studies that investigate the COVID-related impacts on only one individual transport mode, i.e., road (10 studies), air (7 studies), and seaport (9 studies). In addition, three studies address the impacts on two or more transport modes, while six studies discuss the impacts on the four modes or from a general perspective without focusing on a specific transport mode. It can be noted from Table 5 that road freight transport is the most studied mode, while rail freight transport is rarely studied. This may be because road freight transport is the dominant transport mode in most countries compared to other freight transport modes.

#### 4.1.3. Research Methods

Regarding the research methods, the identified literature employs different methods to study the COVID-related impacts. These methods are grouped into four method categories: (1) empirical, (2) quantitative, (3) literature review, and (4) mixed methods. In addition, each method category includes a number of specific methods, as shown in Table 6. The most frequently used method category is the quantitative method (secondary data analysis and regression, forecasting, and mathematical models), followed by empirical methods (case studies, questionnaires, and interviews). Only one study conducts a systematic literature review on the types of crises that affect Europe in the previous 20 years [20]. Some studies employ mixed methods by merging two different methods to improve and validate the overall results [37,48]. For example, Narasimha, Jena and Majhi [37] demonstrate the negative impact of COVID-19 on four Indian seaports using sets of secondary data, i.e., cargo transshipment data of the year 2020. To obtain more insights into the results, they collect the opinions of some experts on the results.

#### 4.1.4. Research Gaps and Trends for Future Research

Reviewing the 36 studies shows a variety of interesting paths for future research as follows:

- Since most reviewed studies focus on the peak period of the COVID-19 pandemic (January–June 2020), more research is needed to analyze the impacts of COVID-19 on each transport mode, starting from the beginning of the so-called “new norm” (July 2020). This will identify the strengths and weaknesses of each transport mode under the different “new norm” policies adopted to reopen society. It is also of great importance to qualify the analysis with some statistics showing how different “new norm” policies affect the KPIs of the individual transport modes. This will support policymakers and practitioners in learning from past events and in formulating better policies for unexpected future pandemics.
- There is a lack of studies investigating the impacts of COVID-19 on freight transport in developing countries. Therefore, further research may evaluate the impacts of COVID-19 on freight transport in developing countries and compare them with those in developed countries. Additionally, it may be relevant to compare different control measures implemented by these countries and to assess the impacts of these measures on the performances of freight transport.
- Many studies report severe pressure exerted by the pandemic on city logistics [6,8,11,27]. Therefore, future research may examine the resilience of specific city logistics initiatives, e.g., smart lockers, collection points, etc., during the early and “new norm” periods of the pandemic, where new buying habits of consumers emerge and will need more resilient and efficient urban logistics. This will help policymakers and practitioners in defining better initiatives and relaxing resisting regulations for possibly occurring waves of the pandemic.
- Some studies propose prediction models for evaluating the impacts of COVID-19 on freight transport performances during 2020 [36,39,54]. Future research may extend these studies by comparing the predicted results with the actual results. Furthermore, it is relevant to develop a better understanding of the main factors that may lead to any deviations between both results. This is expected to guide researchers in developing more robust prediction models for future unexpected pandemics.
- Most studies evaluate the economic impact of the pandemic on freight transport while very few studies study the social and environmental impacts [5,28,38,56]. Therefore, future research may be directed at measuring the social and environmental impacts of the pandemic on individual freight transport modes in general, and urban freight deliveries in particular. Furthermore, existing studies use only traffic data in estimating the carbon emissions from freight transport during the pandemic. Hence, future research may give a complete picture by calculating the reduction in CO_2_ emissions concerned with the utilization of different transport facilities and infrastructure.
- Most studies provide pieces of evidence of the impacts of COVID-19 based on data analysis from a macro perspective [5,6,27,28,29,38,45,56]. Therefore, future research may adopt various methods based on empirical data analysis from a micro perspective to develop more managerial insights by answering several questions of interest, for example:
  1. What are the short- and long-term impacts of the pandemic on different KPIs of logistics companies?
  2. What are the causal relationships among the various impacts of the COVID-19 pandemic?
  3. How do the impacts of the COVID-19 pandemic vary among logistics companies handling different freight or serving different industrial sectors?
  4. How can logistics companies deal with the pandemic and to what extent do support policies from the governments help them to alleviate the impact of COVID-19?
- Further avenues for future research may address developing simulation models to evaluate the long-term effects of different pandemic control policies on freight transport modes. This can have a significant value for policymakers, since the pandemic is still ongoing and will probably have multiple waves caused by new virus variants. In developing models, researchers can greatly benefit from the findings of the literature on the early stage of the pandemic.

### 4.2. COVID-19-Related Mitigation Strategies

In response to the current pandemic, some studies propose various mitigation strategies to minimize the impact of COVID-19 on the different modes of freight transport. By accurately investigating existing literature, we notice a limited number of studies, around 20 studies, discussing mitigation strategies. These strategies include (1) usage of autonomous delivery vehicles (ADVs), (2) deployment of drone delivery, (3) relaxing existing regulations, (4) utilization of mobile warehouses, (5) engagement of large ships, (6) applying quantity discounts, (7) capacity augmentation, and (8) mixed strategies. Table 7 summarizes the mitigation strategies discussed in the literature, and the impacts to be mitigated. As in the previous section, the studies identified here are classified according to the mitigation strategy, transport mode, and the research methodology. In the last subsection, the research gaps, along with future research directions, are elaborated.

#### 4.2.1. Mitigation Strategies

- Usage of autonomous delivery vehicles (ADVs):

ADVs are self-driving electric ground vehicles that travel at a restricted speed, around 5–10 km/h on roadways or sidewalks. Using ADVs as a mitigation strategy can reduce the impact of driver capacity shortage, as ADVs are self-driving vehicles, and enhance the positive impact of emission reduction, as ADVs move at a low speed releasing a small amount of CO_2_. There are six studies that focus on ADVs as a mitigation strategy on road freight transport. Kapser, Abdelrahman and Bernecker [63] investigate user acceptance of ADVs during COVID-19 through the acceptance unified theory. This study is conducted based on real data collected from Germany using online questionnaires. Findings reveal that the user acceptance rate is slightly low, due to a high shipment cost resulting from the expensive technology used in ADVs. Similar to Kapser, Abdelrahman and Bernecker [63], Pani, Mishra, Golias and Figliozzi [67] study the public acceptance of ADVs, but in Portland, USA. Based on a sample of 483 consumers, results indicate that the majority of US consumers, around 61.28%, are willing to pay extra to receive their goods using ADVs. Chen, Demir, Huang and Qiu [10] discuss the idea of food delivery using a traditional vehicle along with robots to serve customers in the last miles. This study shows the efficiency of using these robots in minimizing the contact between the customers and drivers. Besides delivering food products, as shown in the previous studies, Ozkan and Atli [66] present the usage of ADVs in transporting PCR testing samples between hospitals and laboratories. To this end, a mathematical model is presented as a mixed-integer linear programming model (MILP), considering the payload of the ADVs. Using a real case in Istanbul, including 219 hospitals and 23 laboratories, the proposed model shows its efficiency in transporting around 25,000 samples.

Although some studies focus on the usage of ADVs to mitigate the impact of driver shortage capacity, other studies evaluated the usage of ADVs to mitigate the impact of gas emission of traditional vehicles. Li, He, Keoleian, Kim, De Kleine, Wallington and Kemp [64] study the greenhouse gas emission of automated vehicles (last mile) assisted with robots (last 50 feet) used for last-mile delivery. Their results show the outperformance of the automated vehicles with robots over the traditional option (traditional vehicle with human for final delivery) in minimizing the gas emissions.

ADVs are also technically discussed, especially the hardware design. Liu, Liao, Gan, Ma, Cheng, Xie, Wang, Chen, Zhu, Zhang, Chen, Liu, Xie, Yu, Guo, Li, Yuan, Han, Chen, Ye, Jiao, Yun, Xu, Wang, Huang, Wang, Cai, Sun, Liu, Wang and Liu [65] assess the design of the hardware of ADVs, particularly the chassis and autonomous driving-related devices. Based on a real case study containing 25 ADVs, these ADVs are successful to run a total distance of 2500 km, satisfying 676 delivery tasks.

- Deployment of drone delivery

Technically, drones are teleoperated flying machines that do not need continual operator control. In real practice, drones often fly using batteries, which help in carrying goods. These features have attracted the attention of some companies such as UPS and AMAZON to use drones for delivery [78]. This inspires the usage of drones as a mitigation strategy to reduce the impact of driver capacity shortage, improve the impact of delivery performance, as drones are fast transport means, and enhance the positive impact of emission reduction, as drones do not emit CO_2_. Four studies in the literature report the usage of drone delivery as an alternative to road freight transport. Kunovjanek and Wankmüller [69] study the viability of using existing drone infrastructure to deliver COVID-19 viral test to infected people in Australia. This study reveals the viability of using the drone in terms of time and financial measures. However, transport policy needs to be relaxed to permit widespread usage of this service. It is somewhat challenging to establish a legal framework for this service. Alsamhi, Lee, Guizani, Kumar, Qiao and Liu [68] introduce a conceptual framework of integrating blockchain with drone delivery. With the help of a consensus algorithm, the blockchain is fruitful in easing the collaboration among multi-drones by sharing the delivery information among them. Thus, the delivery time is reduced, resulting in an improvement in delivery service. Yakushiji, Fujita, Murata, Hiroi, Hamabe and Yakushiji [71] assess the usage of drones to transport medical supplies in Japan during traffic blockages. Similarly, Quintanilla García, Vera Vélez, Alcaraz Martínez, Vidal Ull and Fernández Gallo [70] investigate the efficiency of using drones as extra support for healthcare logistics in Valencia, Spain. These studies indicate a successful application of drones in transporting medical goods without needing a dedicated infrastructure. Bathke, C, x00Fc, nch, Gracht and Hartmann [72] assess some resilience strategies in the domain of maritime container shipping with the help of 51 maritime experts. The results indicate that picking the low-hanging fruit requires the deployment of drones to improve resilience in the maritime container shipping domain.

- Relaxing existing regulations

COVID-19 prevention and protective measures (e.g., multiple drivers are prevented from using the same truck) increased transport costs. Some countries, such as Canada, increased the maximum hours of service for drivers [60]. In response to air freight capacity shortages, passenger aircraft are granted exemptions from July 2020 to July 2021 to carry freight in the main cabin when no passengers are being carried [59]. To reduce the operating costs of logistics companies, some companies, e.g., in China, exempt logistics enterprises from vehicle tolls and value-added taxes on income due to delivering essential living supplies [8]. The European Union relaxed some rules of the competition law to allow competing companies to cooperate, which might help them overcome capacity shortages or better respond to disrupted supply channels [79].

- Utilization of mobile warehouse

The mobile warehouse is simply a truck that covers a specific geographical location, carrying an inventory of some products to be sold to the customers according to the demand of these products. Utilization of the mobile warehouse as a mitigation strategy mitigates the impact of delivery performance as the mobile warehouse reaches near the customers’ locations. This strategy has not received much attention from researchers, with a single study reported in road freight transport. Srivatsa Srinivas and Marathe [11] are among the first to propose the utilization of mobile warehouses during COVID-19. Using such means can aid B2C e-commerce, which can minimize store congestion during COVID-19.

- Usage of large ships

The main rationale of using this strategy is to satisfy the high demand for goods with a fewer number of ship trips, limiting human movement, and, therefore, control the spread of COVID-19. In addition, using large ships as a mitigation strategy can reduce the impact of container capacity shortage, as using large ships can fix the problem of returning empty containers to the importing companies. This strategy is discussed in the study by Pasha, Dulebenets, Fathollahi-Fard, Tian, Lau, Singh and Liang [73], who develop a mathematical model to determine the service frequency and ship sailing speed and schedules. By using 10 real cases, the proposed strategy demonstrates its efficiency in satisfying food demand while improving the total turnaround profit by approximately 22.94%.

- Application of quantity discount

Due to COVID-19, cargo demand in some routes is disrupted (e.g., demand in some routes exceeds the capacity of the aircraft (hot selling), whereas the demand in other routes is not sufficient to fill the aircraft capacity (underutilized)). This disruption results in a problem, called demand imbalance, or demand shortage for air freight transport. Shaban, Chan and Chung [74] propose a game-theoretic model to make a balance between the hot-selling and underutilized routes. The balance can be achieved by applying a quantity discount in the underutilized routes to attract some demand from hot-selling routes, ultimately leading to fixing the imbalance. To summarize, using this strategy can help to mitigate the impact of demand shortage for air freight transport.

- Capacity augmentation

The main concept of this strategy is that, instead of reserving the normal capacity of the product, the logistics facilities temporarily increase their reserved capacity. This extra reserved capacity will help face disruptions that happen during COVID-19. Aloui, Hamani and Delahoche [75] study this strategy by proposing a two-stage stochastic mixed-integer programming model that helps make some decisions related to location-allocation, inventory, and routing planning. Schofer, Mahmassani and Ng [76] conduct in-depth interviews with Rail Intermodal Freight leaders to figure out how to efficiently enhance the resilience of the U.S. rail industry. The results of these interviews reveal the importance of capacity augmentation in improving the overall freight system performance.

- Mixed strategies

In the previous sub-sections, all the studies focus on applying a single strategy. However, some studies focus on studying more than one strategy. Simić, Lazarević and Dobrodolac [77] apply a fuzzy method to select the best transportation means among drones, ADVs, cargo bicycles, and Postmates for the last-mile delivery during COVID-19. By presenting a real case in Belgrade, results indicate that “postmates” is the best mode, followed by drones and ADVs.

#### 4.2.2. Transport Mode and Research Methods

In this subsection, we classify the studies on mitigation strategies based on transport modes. Table 8 indicates that “road” has been extensively investigated by scholars in 14 studies. On the contrary, “air” and “seaport” modes are barely discussed in the literature, as they only appear in four studies. The road is the most commonly used mode of transport worldwide. Therefore, it is frequently investigated, which is also observed in the COVID-19 impact studies.

The research methods used in the studies of mitigation strategies are also reviewed. For this purpose, these studies are divided into two categories: empirical and quantitative, such that each category is further sub-categorized to other methods, as shown in Table 9.

Table 9 reveals that both the quantitative method and empirical methods are equally used research methods, each one appearing in ten of the 20 studies. Indeed, for quantitative methods, five articles focus on optimization methods using different approaches such as two-stage meta-heuristics algorithm [10], CPLEX [66], deep-learning-based algorithms [65], and recursive route re-composition heuristic [73]. The remaining quantitative methods, including simulation optimization, analytical model, fuzzy method, and game theory, are explored in the rest of studies.

Empirical methods are discussed in 10 out of 17 articles. In particular, four studies use the case studies method. For instance, Li, He, Keoleian, Kim, De Kleine, Wallington and Kemp [64] present a case study to assess greenhouse gas emissions of automated vehicles. In addition, Yakushiji et al. [10] assess a real case study on flying drones to transport medical supplies. The other six studies rely on questionnaires and interviews to investigate user acceptance of ADVs [63,67] and assess the technologies that help increase resilience efficiency at maritime container shipping companies [72]. The limited number of empirical studies indicates that scholars are still faced with the challenges of acquiring and investigating real data. However, the existing empirical studies help to understand the attitudes of people towards the usage of new transportation tools such as ADVs and drones [63,67,71].

#### 4.2.3. Research Gaps and Trends for Future Research Directions

After investigating the 20 articles, which focus on the mitigation strategies, some research gaps along with future research directions are identified as follows:

- There is a low user acceptance of ADVs in Germany [63]. Indeed, ADVs are still immature; therefore, the results presented in these studies are largely premature. Accordingly, more research is required to check the user acceptance of ADVs after the maturation phase. In addition, limited factors, such as age, gender, and citizen income, are considered in evaluating the user acceptance of ADVs [63]. To generalize these findings, more factors, such as delivery distance and time, need to be investigated. Besides Germany, user acceptance of ADVs has only been evaluated in Portland, USA [67]. The investigation is limited to a metropolitan area with small data size, restricting the knowledge of public acceptance of ADVs. In this connection, considering multiple US states with larger data set is imperative to test the public acceptance of ADVs. In addition, making the comparison between ADVs and drones is suggested to see the advantages and disadvantages of each transportation means.
- Robots have been shown to help in minimizing the contact between customers and drivers of traditional vehicles [10]. Here, the only drawback may include overlooking some features of the robots, such as energy consumption and operational cost, which are likely to limit a large-scale deployment of robots. Accordingly, these features should be incorporated in future research to arrive at a better assessment of the applicability of robots.
- Utilization of ADVs with robots is also asserted to minimize greenhouse gas emissions [64]. However, some considerations which are known to affect gas emissions, such as traffic and weather conditions, should be incorporated into future studies to accurately determine greenhouse gas emissions.
- The viability of using existing drone infrastructure, based on time and cost measures, has been verified in Australia [69]. However, many factors, such as legal visibility and opinion of policymakers, have not been considered. For a better verification process, these factors should be investigated in future research.
- Although drones have been used to transport medical supplies in Japan during traffic blockages, it has only been during favorable weather conditions, such as low wind speed and no rain [71]. Therefore, it is imperative to investigate the usage of drones in different weather conditions to further evaluate their applicability.
- Similarly, drones can successfully transport medical goods without the need of specialized infrastructure in Spain [70]. However, this application is accomplished only on a small scale. To enable a large-scale deployment, more investigation is needed.
- The deployment of mobile warehouses has been evaluated by Srivatsa Srinivas and Marathe [11]. Indeed, this study lacks an accurate estimation of product demand. For this purpose, we suggest using data analytic techniques to accurately estimate the product demand. In addition, this study overlooks the dynamic routing and parking optimization of the mobile warehouse. Therefore, investigating this overlooked optimization problem during COVID-19 could be another research direction.
- The deployment of large ships is efficient in satisfying food demand [73]. The main pitfalls of the study include ignoring fluctuations in demand, sea weather conditions, and sailing time of ships, that are typically experienced in real practice. In this connection, it is suggested to develop a stochastic model while considering all uncertain factors to accurately capture this reality.
- Applying quantity discounts appears in the study by Shaban, Chan and Chung [74] while considering deterministic demand. Since demand usually fluctuates, it will be more reliable to consider the stochastic demand while investigating quantity discounts. The planning horizon of the study is quite limited, around one month, which limits the generalization of the gained results. To avoid this situation, it is recommended to study a longer planning horizon (i.e., one year).
- Most studies have presented mitigation strategies for “road” mode, overlooking air and sea modes. Accordingly, more research is required to develop different mitigation strategies for air and sea modes.

### 4.3. Recoverability Measures Related to COVID-19

As stated earlier, the COVID-19 outbreak has had a great impact on freight transport. Therefore, a research stream has studied and evaluated this impact. Then, another search stream has studied measures aimed at counteracting the impact of COVID-19. So, several strategies and policies have been developed to mitigate these impacts. In addition, it is necessary to study the ability of freight transport to recover from the disruption of COVID-19. In this regard, this section summarizes the literature which have studied the measures of recovery of freight transport, during or after COVID-19. Although this stream is still limited and needs further work, there are 15 articles in the existing literature that have assessed various recovery measures using several research methodologies.

#### 4.3.1. Recovery Measures

Few studies tackle the ability to recover in freight transport. However, they adopt different measures of recovery for freight transport, as shown in Table 10. These measures include quality, efficiency, sustainability, capacity, and rate of recovery. The quality as a recovery measure is investigated by Gnap, et al. [80], who consider the quality of transportation infrastructure as fundamental to strengthening the recovery of road traffic and high-speed trains after COVID-19. Likewise, Tardivo, et al. [81] conclude that the quality of the management system is the most important measure for recovery. However, both studies have not given numerical recovery solutions which can be used in the future to judge the resilience of freight transport. van Tatenhove [82] and Munawar, Khan, Qadir, Kouzani and Mahmud [6] adopt the efficiency measure to study the recovery of freight transport in Europe and Australia. Moreover, sustainability measures are used to study the recoverability of freight transport during COVID-19. Guo, et al. [83] adopt performance measurement techniques to study the recoverability of the aviation industry. Four studies have used at least one sustainability aspect. For example, the socioeconomic aspects are used to study the long-term impact and government role to contribute to the recovery from COVID-19 [84]. Additionally, the economic and environmental aspects are adopted to evaluate the recovery of road transport after the usage of plug-in electric vehicles [85]. Bartle, Lutte and Leuenberger [5] and Akyurek and Bolat [86] add the social measure to economic and environmental aspects to study the recovery in air transport. However, very limited research has tackled the capacity and the recovery rates as measures of recovery. Notteboom, et al. [87] study the adaptive capacity of container shipping lines. The authors compare the capacity of the sea shipping transportation to recover in the era of COVID-19 with the capacity used to recover during the economic crisis of 2008 to 2009. They claim that sea shipping is stable during COVID-19 because the shipping liners adjust their fleets when demand drops. Gudmundsson, et al. [88] and Cheong, et al. [89] evaluate the recovery rates of the air transportation profits and demand, respectively. However, using only one single measure to study the recovery is not enough to address the recoverability of any industry. A generic study used an integrated grey decision-making trial to rank the recovery-based sustainability measures for the freight industry regardless of transportation modes

#### 4.3.2. Research Methods and Transport Modes

This subsection discusses the research methods which have been used to study the recoverability of different transportation modes amid COVID-19. As shown in Table 11, 11 studies have adopted several research methods to explore the recoverability of different transport modes for freight transport. Although the number of articles is very limited on this topic, several methods, including empirical, quantitative, and mixed methods, have been used. For example, three empirical methods are adopted: one to create a framework for the future of a freight transport mode [5], one used a scenario-based approach [82], and another one used index-based evaluation techniques to measure the resilience of transportation mode(s) [90]. As for the quantitative methods, comparative analysis [86], forecasting the recovery of demand [88,92] and profit [89], have also been evaluated. The rest of the studies use mixed methods, such as simple data acquisition and experts’ opinions, to assess the current status and comment on the possibility of recovery [84,87]. Nevertheless, data acquisition is not the only mixed method used; the analytical hierarchy process has also been used to integrate the economic and environmental criteria to evaluate public transportation systems [5,85].

On the focus of transport modes, Table 11 shows that all the transport modes are almost equally studied, as each transport mode is studied by around three to four studies. This observation is contrary to the observation noticed in the impact and mitigation parts.

#### 4.3.3. Research Gaps and Trends for Future Research

Although the COVID-19 pandemic has caused severe disruptions to the freight transport sector, the number of articles does not reflect the criticality of these disruptions. Additionally, the 11 studies examined here can be considered as an initial step towards the recoverability of freight transport, and some future directions can be recommended as follows:

Although there is no numerical evidence that the air transport mode is the most affected amongst the other modes, lockdown of international borders has significantly affected air transport. Most of the studies have focused on the amount of impact, but not many studies tackled the ways to recover after the lockdown. Therefore, more future work is still needed to enhance the recoverability of the air freight transport mode, not only for the post-COVID era but also for any unexpected long-term disruptions.

The continuous mutation of COVID-19 causes huge disruption in freight transport domestically and internationally. Therefore, further studies are suggested to study the recoverability of freight transportation and the way to improve the resilience of each mode is a great challenge to be considered.

Despite the fact that the existing studies have suggested different measures for recovery, a recovery index that uses multiple measures is required to evaluate the recoverability of freight transport. The recovery index may be developed either by freight transport modes in total or for each mode separately.

## 5. Conclusions

The existing review studies lacked a detailed analysis regarding freight transport during COVID-19. Therefore, this study contributed by conducting a comprehensive review in the domain of freight transport to identify some research gaps and propose future research directions. To do so, 68 studies were collected and analyzed using scientometric and systematic review approaches.

The scientometric analysis revealed that: (1) the combination of China, Hong Kong, and India published the highest number of papers on freight transport during COVID-19, whereas authors from Canada and the United States had the highest citations worldwide; (2) *Sustainability* (Switzerland) and *Science of Total Environment* were the top-cited journals, while the *Canadian Journal of Agricultural Economics* was the most influential journal in this research area. Lastly, the analysis of the keywords showed that “COVID-19”, and “Freight transport” appeared frequently, reflecting the focus of researchers on these areas. Meanwhile, some keywords such as “Airline industry” and “Cargo” were rarely used, indicating the need for future research in these areas. The systematic review was carried out by categorizing the studies into three main themes, including impact-related studies, mitigation strategy-related studies, recoverability-related studies. The results reveal that the most studied theme was related to the impacts of COVID-19, followed by mitigation strategies, and then recoverability. Each theme was discussed alongside types and different transportation modes (i.e., road, air, and sea) and solution methods adopted.

Our review showed that 36 studies reported several impacts of the COVID-19 pandemic on freight transport. These impacts were categorized into five main groups, which included demand for freight transport services, transport capacity, operating cost, delivery performances, and carbon emissions. Surprisingly, not all reported impacts were negative. Negative impacts included, for example, reduced demand for transport services, higher operating costs, transport capacity shortages, delayed delivery, and difficulties in finding parking areas. On the contrary, some studies reported some positive impacts, such as increased availability of truck drivers and lower operating costs, increased demand for transport services, tax reductions on the service of logistics companies, fewer traffic jams, increased efficiency, and fewer emissions. Most of these studies discussed the demand-related impact, followed by impacts on operating transport, transport capacity, delivery performance, and carbon emissions. Regarding the transport mode, road freight transport and seaports were the most evaluated modes, followed by airfreight transport, while rail freight transport was rarely addressed. Regarding the employed research methodologies, the most frequently used methods were secondary data analysis, followed by questionnaires and interviews.

After reviewing 20 studies focusing on mitigation strategies, we observed eight types of mitigation strategies, including (1) usage of autonomous delivery vehicles (ADVs), (2) deployment of drone delivery, (3) relaxing existing regulations, (4) utilization of mobile warehouses, (5) engagement of large ships, (6) applying quantity discounts, (7) capacity augmentation, and (8) mixed strategies. It was noticed that the most studied strategy was the usage of ADVs with six studies, whereas the least studied strategies were strategies 4 to 8, with each strategy appearing in one or two studies. After that, the studies were classified based on the transport mode, with the most frequent transport mode in 14 studies being the road. The least frequent modes were air and seaport, each mode occurred in one or two studies. The last classification was based on the research method, such as empirical methods and quantitative methods. This classification indicated that both quantitative methods and empirical methods were equally used, such that each one appeared in ten studies.

In light of the undesirable impacts of pandemics and the mitigation strategies to overcome these impacts, it was necessary to study the recoverability of freight transport in its three modes. Nevertheless, only 15 studies covered the recoverability topic, discussing six recovery measures, such as quality, efficiency, sustainability, performance, capacity, and rate of recovery. Most of these studies, 5 out of 15 studies, focused on sustainability as a recovery measure, while little attention was paid to the rest of the recovery measures. In addition, the recoverability studies discussed all the transport modes with the same attention, as each transport mode was studied by an average of six studies. The recoverability studies were discussed using different research methods, such as empirical methods, quantitative methods, and mixed methods. It was observed that the most studied method was the mixed method (i.e., correlation analysis and data acquisition), which comprised approximately 8 out of 15 studies, while the rest of the methods received almost the same attention from scholars, from two to four studies for each method.

**Figure 6:**
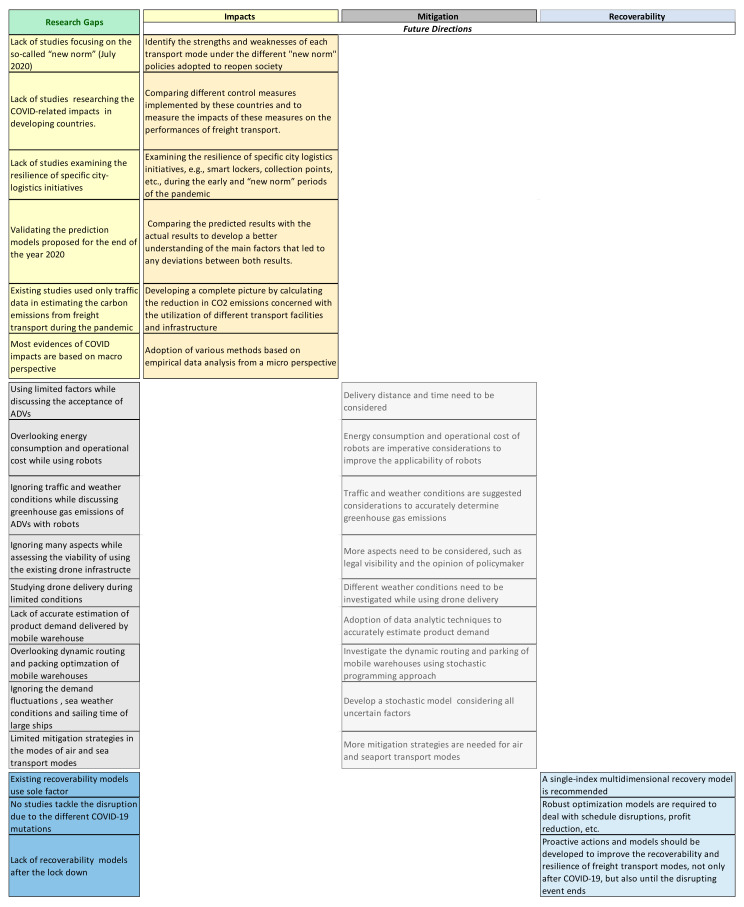
Research gaps and future directions matrix.

After reviewing the studies on the different themes, some future research directions were highlighted. Regarding the impact theme, studying the impacts of COVID-19 in developing countries was suggested. Focusing on the mitigation theme, more mitigation strategies were needed for air and seaport transport modes, as their related strategies were limited. Additionally, investigating dynamic routing and parking of mobile warehouses were suggested. Based on the recoverability theme, it was recommended to develop robust optimization models to deal with schedule disruptions and profit reduction. More detailed research gaps and future directions are represented in Figure 6.

Although the novelty of this study has been highlighted, it is, nevertheless, important to highlight some of its limitations. For instance, some scientometric analyses, such as the most prolific/active authors, were not provided to avoid making the study too long. Similarly, in the systematic review, we overlooked studies on freight transport before COVID-19. It might be interesting research to study freight transport before and during COVID-19.

## Figures and Tables

**Table 1:** Summary of results of some selected scientometric analyses.

Analysis	Total Number of (---)	Threshold Criteria Minimum Number of			Number of (---) Meet the Threshold	Number of Connected (---)
		Occurrence	Documents	Citation		
Co-occurrence of (keywords)	604	3	--	--	42	42
Co-authorship between (countries)	40	--	2	0	18	14
Citation of (sources)	36	--	1	0	36	13

**Table 2:** Top used keywords.

	Keyword	Links	Occurrences
1	COVID-19	38	52
2	Freight transport	20	14
3	Supply chains	8	10
4	China	25	9
5	Epidemic	19	8
6	Viral disease	26	8
7	Sustainability	10	8
8	Air transportation	15	6
9	Logistics	14	5
10	Ships	9	5
11	Transportation policy	15	5
12	Airline industry	9	4
13	Cargo	12	4
14	Human	15	4
15	Transportation system	9	4

**Table 3:** Top cited sources.

	Source	Documents	Citations	Normalized Citations	Avg. Citations
1	Canadian Journal of Agricultural Economics	2	367	9.1039	183.5
2	Transportation Research Interdisciplinary Perspectives	3	90	3.7687	30
3	International Journal of Advanced Manufacturing Technology	1	79	1	79
4	Transport Policy	7	49	8.7427	7
5	Sustainability (Switzerland)	10	39	5.4223	3.9
6	Science of the Total Environment	1	37	6.6017	37
7	IEEE Engineering Management Review	1	30	0.7442	30
8	International Journal of Logistics Research and Applications	1	20	0.4961	20
9	Ocean and Coastal Management	1	10	1.7842	10

**Table 4:** Categorization of different impacts of COVID-19 on freight transport.

Impacts of COVID-19	Studies per Impact	References
Demand for freight transport services	24	[5,6,7,8,9,11,27,28,29,30,31,32,33,34,35,36,37,38,39,40,41,42,43]
Capacity shortage	10	[5,6,32,34,35,44,45,46,47,48]
Operating transport cost	13	[5,6,7,8,20,27,31,32,34,35,49,50,51,52]
Delivery performance	9	[8,9,32,47,48,53,54,55]
CO_2_ emissions	4	[5,28,38,56]

**Table 5:** Classification of COVID-19-related impact studies based on transport mode.

Transport Mode (s)	Number of Studies per Transport Mode (s)	References
Road	10	[11,27,29,30,35,41,42,43,46,56]
Air	5	[5,6,34,36,38]
Seaport	8	[7,9,33,37,39,40,52,54]
Air and road	1	[28]
Road and rail	1	[45]
Road, sea, and rail	1	[20]
All modes (General)	6	[31,32,44,48,49,55]

**Table 6:** Classification of studies on the impact of COVID-19 based on research method.

Main Method Category	Number of Studies per Main Method Category	Specific Method	Number of Studies per Specific Method	References
Empirical methods	8	Case studies	3	[30,34,44]
		Questionnaires and interviews	5	[32,35,44,49,53]
Quantitative methods	21	Secondary data analysis	15	[5,6,27,28,29,38,40,41,43,45,47,50,51,52,56]
		Regression models	4	[7,8,9,33]
		Forecasting models	3	[36,39,54]
		Analytical models	4	[11,31,42,55]
Literature review methods	1	Systematic review	1	[20]
Mixed methods	2	Questionnaires and second data analysis	1	[37]
		Review and second data analysis	1	[48]

**Table 7:** Mitigation strategies for minimizing the impact of COVID-19.

COVID-19-Related Mitigation Strategies	Impact to Be Mitigated	Studies per Strategy	References
Usage of autonomous delivery vehicles	Driver capacity shortage, emissions	6	[10,63,64,65,66,67]
Deployment of drone delivery	Driver capacity shortage, delivery performance, emissions	5	[68,69,70,71,72]
Relaxing existing regulations	Capacity shortage and operating cost	3	[8,59,60]
Utilization of mobile warehouses	Delivery performance	1	[11]
Engagement of large ships	Container capacity shortage	1	[73]
Application of quantity discounts	Demand for freight transport service	1	[74]
Capacity augmentation	Driver capacity shortage, delivery performance	2	[75,76]
Mixed strategies	Driver capacity shortage, delivery performance, emissions	1	[77]

**Table 8:** Classification of COVID-19-related strategies studies based on transport mode.

Transport Mode (s)	Number of Studies per Transport Mode (s)	References
Road	14	[8,10,11,60,63,64,65,66,67,68,69,70,71,75,76,77]
Air	2	[59,74]
Seaport and waterways	2	[72,73]

**Table 9:** Classification of studies on COVID-19-related strategies based on research method.

Main Method Category	Number of Studies per Main Method Category	Specific Method	Number of Studies per Specific Method	References
Empirical methods	10	Case studies	4	[59,64,70,71]
		Questionnaires and interviews	6	[8,60,63,67,72,76]
Quantitative methods	10	Optimization	5	[10,65,66,68,73]
		Simulation optimization	2	[69,75]
		Analytical model	1	[11]
		Fuzzy method	1	[77]
		Game theory	1	[74]

**Table 10:** Summary of recovery measures.

Recovery Measure	Study per Measure	References
Quality	2	[80,81]
Efficiency	3	[6,82,90]
Performance	1	[83]
Sustainability	5	[5,84,85,86,91]
Capacity	2	[87,90,92]
Recovery rate	3	[88,89,90]

**Table 11:** Classification of research methods based on the transportation mode.

Main Method Category	Number of Studies per Main Method Category	Specific Method	Transportation Mode			References
			Road and Railways	Seaports and Waterways	Air	
Empirical methods	3	Framework study			✓	[5]
		Scenario-based research		✓		[82]
		Index-based evaluation	✓			[90]
Quantitative methods	5	Forecasting	✓	✓	✓	[88,89,92]
		Comparative analysis	✓	✓	✓	[86,91]
Mixed methods	8	AHP multi-criteria decision making	✓		✓	[83,85]
		Correlation analysis	✓			[80]
		Data acquisition	✓	✓	✓	[5,6,81,84,87]

## Data Availability

The search queries used in Scopus during the study are available from the corresponding authors on reasonable requests.
